# Psychopharmacology Today: Where are We and Where Do We Go From Here?

**DOI:** 10.4103/0973-1229.58816

**Published:** 2010

**Authors:** Thomas L. Schwartz

**Affiliations:** * *Associate Professor of Psychiatry, SUNY Upstate Medical University, Syracuse, NY 13210, USA*

**Keywords:** *Psychopharmacology*, *Pharmacodynamics*, *Psychiatric outcomes*, *Prescribing practices*

## Abstract

Since the 1950s we have had the same three neurotransmitters to work with to treat depression, one transmitter for psychoses, three for anxiety. We have developed newer drugs that are more tolerable, but we have not developed drugs that are better in efficacy. The last 50-60 years should be considered the decades that allowed us to treat a greater number of patients with safer and more tolerable drugs. We have also decreased stigma and allowed primary care clinicians to become more comfortable treating the mentally ill. We clearly treat more patients than before, and sometimes are now accused of over-prescribing wantonly as our drugs are safer. Without any clear blockbuster new drug ready to be added to our armamentarium, what can we do as psychopharmacologists today, and tomorrow, to obtain better results? This introductory manuscript will attempt to provide an overview of ideas so that an adept, well-rounded clinician might be able to obtain better outcomes despite using neurotransmitter pharmacodynamics that have been around since the 1950s. Finally, I will comment on the psychotropic pipeline, which may be added to our armamentarium in the future.

## Introduction

Things seem fairly straightforward. We have a limited amount of neurotransmitters and receptors to work with in terms of getting our mentally ill patients better. Facts are facts. I will use major depressive disorder as a hallmark illness to make many of my points throughout this manuscript. The discovery and initial use of the MAOi and tricyclic antidepressants was a huge turning point in the world of psychiatry. We finally had reliable, reproducible methods to treat our patients who could be studied the way ‘medical illnesses’ are studied and treated. Our antidepressants and anti-manic agents are studied in a way that the average patient must achieve average (50%) efficacy over placebo in an average number of patients in the study (Thase, 2003; Keck, 2009). It is only 30% for schizophrenia and mania (Canuso, 2009). Our drugs are average at best, and we have been using the same set of neurotransmitters and receptors to beat depression and other illnesses since the 1950s. We are not making better medications from an effectiveness point of view, but clearly have made safer, more tolerable agents. We have been able to get even more patients, an average amount better. Most guidelines suggest we obtain remission, 100% improvement, or wellness for our patients, but our best-studied monotherapies obtain that less than one-third of the time (Zajecka and Goldstein, 2009).

Given our ‘old’ and ‘average’ pharmacodynamics used since the 1950s, what can we do better for our patients? This manuscript will discuss some of the author’s best practices and ideas, as unfortunately, there is not a remarkable evidence base for getting all patients to remission.

## Basic Psychopharmacology Principles

### Dosing

Every psychotropic has a simple dose-response curve. One must dose higher than the minimum effective dose, or the drug will not work. However, most of our psychotropics often carry official wording that ‘there is no additional benefit’ from dosing higher than the minimal effective dose. While lecturing large groups, I often ask psychiatrists if they feel this is true, or if dosing higher within regulatory guidelines is worth it? The unanimous conclusion is that higher dose monotherapies work better, but come with the cost of greater side-effect burden, poorer compliance, and greater expense.

Why is it that most of us feel higher doses work better, but no one can officially prove this? First, the FDA in the U.S. only requires agents to be better than placebo with improvements of 20-50% depending upon which mental disorder is being studied. Second, sample sizes are usually 300 subjects and these are powered statistically to show that an agent needs to be better than placebo with improvements of 20-50% only. As our agents are fairly effective, rating scales may be lowered 50% after initial dosing of the drug, leaving little room for improvement as doses are escalated. There are fewer patients remaining in the study with significant symptoms where 50% more improvement can even occur. Leaders in the field of depression research suggest that studies with 1000-2000 subjects may be needed to truly show that dose escalation statistically works (Schwartz, Thase, Storman, 2006; Cohen, 1977). In the meantime, we are left with sample sizes equal to one: our patient at hand. Clinically, we should aim for the minimum therapeutic dose, wait four to six weeks and evaluate. If there is no effect, dose to the mid-range per regulatory guidelines for four to six weeks. If there is still no effect, aim for the highest dose allowed and wait several weeks. If there is no effect even now, at least there is proof that a full dose and full duration was used.

### Duration

What is a good duration for an antidepressant monotherapy trial? Some authors suggest that if there is no partial response within the first two weeks then remission is doubtful (Szegedi *et al*. 2009). Others, and the NIMH-sponsored STAR*D (Trivedi, Rush, Wisniewski, *et al*. 2006) trial, suggest that 12 weeks at moderate to high dosing is really needed as some patients respond early and some later. Clearly, there is room for the psychopharmacologist again to treat patients on an individual basis and to vary duration as needed.

As far as the brain and our neurons are concerned, our antidepressants obviously change transmitter levels almost immediately but we rarely see remission within hours. After a few to several weeks of treatment, we often can see receptor down regulation or build-up in neurotrophic factors. It seems that our antidepressants work by changing neuronal gene activity to facilitate the production of new proteins (neurotrophic factors), which takes four to six weeks (Stahl, 2008; p521-523). So pharmacodynamically, this timeframe is a good duration. What about the sedatives for anxiety, they work within one dose or a few days? They optimize ion channels directly and work quickly.

Antipsychotics? They seem to work within three to four days for mania and one to two weeks for psychosis from schizophrenia. The dopamine receptor blockade here likely diminishes dopamine neurocircuitry tone to provide effectiveness which is moderate in course (Stahl, 2008; p329-332). Perhaps duration of treatment should depend on the agent and the mental disorder.

### Switching

If a clinician can assure a good dose and duration as above, and there is no response at all, then a switch to a new agent should occur. In fact, certain authors might suggest that if an initial monotherapy provides only a partial response from 0-30% better, it should likely be abandoned and a switch to a new monotherapy should occur (Zajecka and Goldstein, 2009). The new drug should affect a different dynamic mechanism. In the case of depression, switching from SSRI to SSRI to SSRI makes little pharmacodynamic and clinical sense. If the response is greater than 30%, then an augmentation/combination approach should occur (Zajecka and Goldstein, 2009). There is almost no conclusive data to support multiple switches or multiple combinations, so oftentimes this is at the discretion of the clinician. We also know that if a drug is not working over many weeks, it probably will not start to work months later, so switching agents is often the prudent choice.

## Advanced Psychopharmacology Principles

Augmenting refers to adding a non-approved agent to boost clinical effectiveness (e.g. thyroid hormone added to tricyclic antidepressant for depression), and combining suggests that a clinician add two approved agents (e.g. bupropion added to sertraline for depression) together instead. The net effect is polypharmacy in order to obtain better outcomes. Again, in the depression world, only one third of patients obtain remission on a monotherapy (Trivedi, Rush, Wisniewski, *et al*. 2006). Therefore, we either need to use the strategy above and maximize monotherapy then switch agents, or add products together. At a large scale national event, I asked a large audience of psychiatrists how many drugs their usual depressed patient is prescribed. The answer was ‘two or more,’ routinely (Schwartz and Rashid, 2007). We have few approvals for combined medications like: amitryptiline-perphenazine for depression and olanzapine-fluoxetine for depression, but most of the time we prescribe ‘off-label’ where there is little to no evidence-base with statistically powered analysis to support our polypharmacy strategies- which seems to be the standard of care for resistant illness.

### Rational Polypharmacy

I’d like to go back to some simple rules offered by a key author in the area of psychopharmacology, Stephen Stahl (Stahl, 2000). When prescribing and combining medications consider the following:

**Figure 1:**
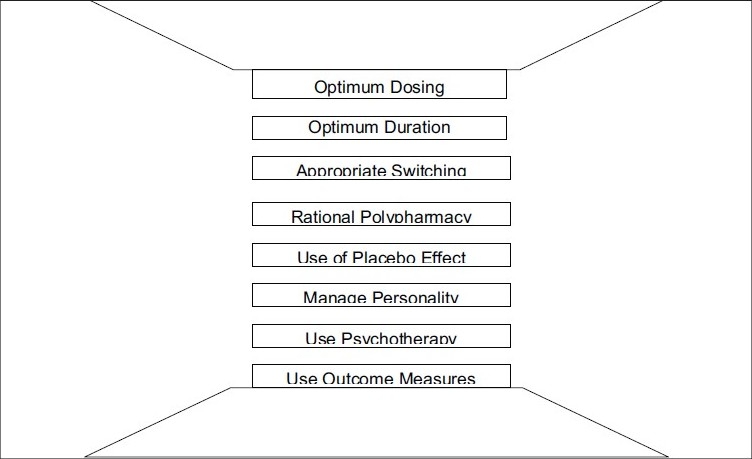
Advancing psychopharmacology and its outcomes requires several skill sets

1. Aim for full remission
2. Use and combine medications that utilize different therapeutic mechanisms of action
3. Continue your education and push your envelope to learn new techniques
4. Monitor for side-effects and establish treatment intolerance vs. resistance
5. Create ‘win-win’ situations where you combine drugs for better effects but also cancel each other’s side-effects
6. Diagnose and treat all comorbidities
7. “Be the student of your patient’s life.” Take a full history, be thorough and make accurate assessment per DSM, dynamics.

Essentially, we have to become excellent at using the tools that we currently have. If monotherapies are average, and there is no ‘cure-all’ drug in the research pipeline, we will have to understand the brain and our patients better and then utilize rational polypharmacy techniques. This does mean memorizing what each agent does to the brain including downstream changes in the brain’s functioning. We may even need to memorize certain genetic terms and findings and neuroanatomic terms and findings to better treat our patients. This requires a certain ‘work ethic’ on the part of the clinician. As you will see, Stahl’s seven items above require hard work, diligence, effort and aggressiveness on the clinician’s part not to cut corners and settle for partial responses. It also challenges us to think multi-dimensionally as far as prescribing multiple medications simultaneously, managing side effects, cost, psychological treatment resistance, checking labs, writing progress notes, and attending CME events.

## Beyond Psychopharmacology

As we have been using the same transmitters and above clinical principles for some time, the newest idea would be to conceptualize what we are doing with regards to fine tuning neurocircuits as noted above. How might a future psychopharmacologist operate? In order to determine what neurocircuit is dysfunctional in ADHD, as an example, we might order a set of genetic tests to determine if there is over, or under, processing of norepinephrine or dopamine, and select an agent accordingly. We might also order a functional neuroimaging study to attempt to decide what brain area is under, or over, functioning. If we know this, we might know what transmitter and receptor complexes operate there and choose a medication accordingly. As we are at least several years away from this, what else can we do now to improve patient outcomes? This section discusses non-psychopharmacological approaches that might boost psychopharmacologic outcomes.

### Placebo effect

Use it! In the world of depression/anxiety this effect is now escalating above 30-40% in randomized trials (Walsh, Seidman, Sysko, Gould, 2002; Rutherford *et al*., 2009). This is likely because we prescribe more psychotropics than ever before. As these drugs are safer, we prescribe more. Our patients, their family and friends take psychotropics, and our patients see television advertisements, stigma drops and we prescribe more. Patient expectations are that these drugs are safe and work well which is a breeding ground for a linearly increasing placebo effect. How can one capitalize on this effect? I offer the following:

- *Build rapport, spend time with your patients*: Their confidence in you will boost your drug’s placebo effect. If they believe in you, they will believe in what you prescribe.
- *Use psychotropics you believe in*. If you have a positive attitude towards the drug you are about to prescribe, patients pick up on it.
- *Use psychotropics the patient believes in*. If the patient comes with a request for a certain medication, even if it is not your optimal choice, but is a reasonable one, they may be more likely to respond to it.
- *Be book smart and know your facts*: Patients will ask you about the epidemiology and etiology of their illness and how the medications may help. If you know your facts and answer their questions directly, you will likely get a better placebo response. Again, this likely increases rapport and patient confidence. If you ignore or minimize these patient questions, then I suspect that compliance and outcomes will suffer.
- *Utilize informed consent*: Explain to your patient that the drug is approved and studied for their condition, be honest and thorough about side-effects. Learn how to describe the drug’s mechanism of action and what it may be doing to help the depressed, anxious, or manic brain. Instead of essentially stating, “take this once a day and see me in four weeks”, giving the patient a better rationale and reason for the prescription will (a) build rapport- [see item (i) above]; and (b) likely have the patient stay on the medication longer, which allows a greater chance of placebo response and/or a true response.

### Understand and recognize personality disorder

As psychopharmacology grows in popularity and the 15-minute ‘med management’ session every 30-90 days becomes the norm, I think we become less adept at ‘reading our patients’ and truly understanding them. We are pretty good at getting the DSM-IV (American Psychiatric Association, 2000) Axis I conditions correct in these short visits and we should be very good at monitoring symptoms and dispensing medications. I think we are often foiled when we see a bipolar patient, for example, and prescribe all sorts of mood stabilizers to no avail. After a few years of 15-minute visits, the age old pattern of borderline personality and affective dyscontrol emerges, and we see why our bipolar patient is no better on our medications. Unfortunately it takes years for us to see these patterns as our visits or sparse in nature and symptom-based. If you do not have the luxury to see your patients more often and for longer sessions, you must liaison with their weekly psychotherapist in order to enhance your diagnostic abilities in the Axis II area. Being realistic about Axis II outcomes from Axis I medications might actually direct you to be more conservative with medications and more aggressive in psychotherapy. A referral for dynamic or dialectical-behavior therapy in these instances might yield better results and has the evidence base to support referral to a specialist in these techniques. (Gregory, *et al*. 2008; Lynch, *et al*. 2007)

### Psychotherapy

As noted above, the art of practicing psychotherapy in an organized manner seems to be waning in both training and practice. There are many reasons for this shift towards ‘med management only’ practices and skill sets, but it seems we are using less of these skills all the time. It is also true that we are ‘always doing psychotherapy’, even in shorter medication visits. It is likely impossible to conduct manualized CBT, DBT, IPT or dynamics, but psycho pharmacologists should never lose sight of, or their skill set of core psychotherapy techniques (motivation, empathy, openness, collaboration, warmth, positive regard, sincerity, providing corrective experience, catharsis, establishing goals, establishing a time limited relationship, establishing that patient effort is needed to succeed) which are common to all psychotherapy styles and common amongst good psychotherapists (Greenberg, 2004). It is quite simple to create an actual, or mental, checklist to follow during session, making sure that these techniques are used quantitatively and qualitatively. This guarantees that supportive psychotherapy occurs, even in a short session. This application, which takes only a fraction of a medication visit, in theory, may promote greater placebo response, real drug response, and may promote better medication adherence and outcomes.

### Outcome measures

Almost every other branch of medicine utilizes outcome measures. Everywhere else, blood pressure and weights are gathered routinely. Beware that this age is likely upon us psycho pharmacologists now that it is well recognized that many of our medications induce weight gain and metabolic illness. Routine blood glucose levels help the diabetologist obtain better compliance and diabetic outcomes. TSH levels help the endocrinologist obtain a euthyroid state. As we do not have a blood pressure cuff or laboratory analysis for mental illness, we must rely on patient rated or clinician rated scales. Data would suggest that psycho pharmacologists who use scales routinely in practice get better outcomes in certain disorders (Zimmerman, McGlinchey, Chelminski, 2009). Is this because better psycho pharmacologists know the value of, and use scales to their advantage, perhaps? More likely, it is that routine use of rating scales allows the clinician to see that full remission is not present, and it forces us to treat patients more aggressively by increasing dose, maximizing duration, adding other medication or psychotherapy until remission occurs.

There are simple self-report scales for almost every disorder. These can be filled out in waiting rooms and scored by secretaries. In the age of the electronic record, patients can enter scales into kiosks or laptops in waiting rooms so that scores are at the psychopharmacologist’s fingertips and automatically stored in databases that can be graphed later to show response, remission, or pending relapse. If you conduct medication visits every 90 days or more, patients can be trained easily to take a survey at home, score it and contact you if the scale is above a cut off for relapse. Scales can be mailed out as appointment reminders or completed early in the waiting room, so as not to take up valuable appointment time. In our practice, we find that when patients walk into our offices and we have already been armed with their scores, we know if we are in remission and have to work on maintenance, or are at a partial response and have to work towards remission. As symptoms are concrete, ‘yes or no’ in nature, and we have seen them courtesy of the scale, we often have more time to build rapport, use core therapy techniques, give better informed consent and bolster compliance.

### Future directions, and psychopharmacological agents, which hold promise

I offer the following with regard to the pipeline and what will ideally become available to us:

For schizophrenia, we might expect to see the decade of glutamate. Specifically, NMDA receptor antagonists could be used to slow down the progression of schizophrenia by reducing neurotoxicity in the CNS. This would have to be balanced well though, as too much blockade could actually cause more psychosis as is seen with phencyclidine intoxication. Using presynaptic metabotropic glutamate receptor agonism might also be able to lower psychotic symptoms. The glutamate circuitry is complex and difficult to manipulate as too little, or too much, can make patients worse in schizophrenia and even anxiety disorders. (DeBartolomeis *et al*. 2005) For depression, triple reuptake inhibitors (SNDRI’s) might be developed, which would allow us to cut down on polypharmacy (Liang, 2008). I suspect that atypical antipsychotics will continue to be released and used for schizophrenia, bipolar illness, and depression; and novel epilepsy medications will continue to be studied in bipolar populations. Finally, novel pharmacodynamic combination drugs like the serotonin-2c blocking, melatonin stimulating, agomelatine, may offer us a unique approach as an antidepressant as well (Bourin and Prica, 2009).

## Concluding Remarks

The premise of this paper is that we have been using the same technology and neurotransmitter systems to treat mental illness since the 1950s. We have made medicines safer, treated more patients, but continue to do an average job as far as outcomes are concerned, with monotherapies. As there is no sweeping cortico-suppressing, brain derived neurotrophic factor enhancing, triple monoamine stimulating, glutamate-dopamine balancing universal blockbuster psychotropic due out in the next 5-10 years, we have to find a way to obtain better outcomes. We must use the ‘average’ medicines at hand; combine them skillfully and aggressively with each other and with non-pharmacologic approaches in order to have excellent outcomes more often than not. As newer agents become available, they should be assessed not only for safety and efficacy, but also for their ability to treat depression in a novel manner. I do think we have entered the era of pharmacogenetics and functional neuroimaging from a psychiatric point of view, but until these are fine tuned and put in our hands, we must be better at using what we have available.

### Take home message

Use approved medications. Dose fully and for adequate durations. If remission is not achieved, combine medications based on the evidence base, clinical rationale, or pharmacodynamic theory. Do not lose the skills of a good psychotherapist and learn to incorporate rating scales into practice.

### Conflict of interest

The author receives funding from the following entities for research grants, consulting, or corporate speaking: Astra-Zeneca, Bristol Myers Squibb, Cephalon, Cyberonics, Forest, GlaxoSmithKline, PamLab, Pfizer, Wyeth.

### Declaration

This paper is the original work of the author and has not been submitted for publication previously, nor is it being reviewed by any other entity.

## Questions That the Paper Raises

1. Are psychotropics mildly, moderately or extremely effective?
2. Are certain psychotropics better than others?
3. What can a psychopharmacologist due to achieve better patient outcomes?
4. Are single medication treatments better than combinations or polypharmacy?
5. Can we use genetic testing, functional neuroimaging to pick out the best psychotropics?
6. Should we raise the bar for treatment outcomes in mental disorders?

## About the Author


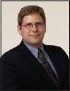
 *Thomas L. Schwartz, MD, received his medical degree from and completed his residency in Adult Psychiatry at the State University of New York (SUNY) Upstate Medical University in Syracuse, New York. Dr Schwartz is currently Associate Professor of Psychiatry, Director of Adult Outpatient Services, and Assistant Director for psychiatric residency training at SUNY Upstate Medical University, where he also directs the Depression & Anxiety Disorders Research Program. Active on many committees at SUNY, he also provides direct resident supervision, lectures in several courses, and directs and organizes continuing medical education events for the psychiatry department. Dr Schwartz also maintains a private practice and consultation practice.*

He is a member of the American Psychiatric Association and has been honored with their Nancy Roeske, MD and Irma Bland, Certificates of Recognition for Excellence in Medical Student and Resident Education from the American Psychiatric Association.

Dr Schwarz has served as principle investigator on many clinical trials; his clinical and research interests include treatment-resistant depression and anxiety, psychosomatic illness, adult psychopharmacology, and antidepressant augmentation for efficacy and tolerability. He is the editor of Depression: Treatment Strategies and Management, 2nd Ed.
